# Effects of different physical therapy training protocols on patients with idiopathic scoliosis: Short-term results

**DOI:** 10.1371/journal.pone.0334713

**Published:** 2025-10-17

**Authors:** Haohan Lu, Min Li, Naizhen Wang, Guobiao Ye, Feilong Zhu, Mingling Guo, Cheng Zeng

**Affiliations:** 1 Fujian Children’s Hospital (Fujian Branch of Shanghai Children’s Medical Center), College of Clinical Medicine for Obstetrics and Gynecology and Pediatrics, Fujian Medical University, Fujian, China; 2 Department of Rehabilitation, Fuzhou Second General Hospital, Fujian, China; 3 College of Physical Education and Sports, Beijing Normal University, Beijing, China; Nippon Medical School, JAPAN

## Abstract

**Background:**

Idiopathic scoliosis (IS), a three-dimensional spinal deformity common in adolescents, can be treated with numerous approaches, including Schroth, Spiral stabilization (SPS), and core exercises, to improve spinal alignment and health. However, few studies have shown the superiority of one treatment over another.

**Objective:**

This retrospective cohort study was designed to evaluate the efficacy of three distinct physical therapy protocols in managing IS among adolescents aged 6–18 years with Cobb angles ≤40°.

**Methods:**

A total of 61 patients were allocated to one of three therapeutic groups. Each group received interventions twice weekly for 10 sessions. Primary outcomes were assessed by measuring changes in the Cobb angle, whereas secondary outcomes included evaluations of pain (visual analog scale, VAS), the angle of trunk rotation (ATR), trunk aesthetic clinical evaluation (TRACE), radiographic parameters (Alignment of the C7 plumbline (C7PL), clavicle angle, and pelvic obliquity), and quality of life (the Scoliosis Research Society-22 questionnaire,SRS-22).

**Results:**

The findings revealed significant within-group improvements in the Cobb angle, pain, and trunk rotation for all interventions (p < 0.05). Notably, between-group comparisons indicated that both the Schroth and SPS modalities resulted in greater reductions in the Cobb angle and trunk rotation than core stabilization exercises did (p < 0.05). Moreover, SPS resulted in superior enhancements in the clavicle angle and dynamic postural control, whereas Schroth yielded significant improvements in C7PL alignment.

**Conclusion:**

This short-term study underscores the relative effectiveness of the Schroth and SPS interventions in managing three-dimensional spinal deformities and enhancing dynamic postural control while recognizing the limitations of core stabilization exercises for scoliosis.

**Clinical trial registration:**

This retrospective analysis of a clinical trial has been registered in the Chinese Clinical Trial Registry (ChiCTR2500096077) https://www.google.com/search?client=firefox-b-d&q=ChiCTR2500096077.

## 1. Introduction

Idiopathic scoliosis (IS) is a prevalent three-dimensional structural deformity of the spine characterized by an abnormal curvature coupled with torsional distortion [1–3]. Its definition varies among studies, but IS generally refers to a Cobb angle that exceeds 10° [4,5] excluding other identifiable causes, such as congenital or neuromuscular disorders [6,7]. Pathologically, IS is frequently perceived as a consequence of imbalances in the growth and development of the spinal structure and its associated musculature [8,9]. The term “idiopathic” highlights the unclear etiology of this condition, although multiple hypotheses, ranging from genetic predisposition to biomechanical imbalances, have been proposed.

IS predominantly affects adolescents during growth spurts. Adolescent idiopathic scoliosis (AIS) is the most prevalent subtype and accounts for more than 90% of all cases of spinal scoliosis [10]. In addition to being a three-dimensional deformity, IS significantly impacts patients’ quality of life (QoL) and psychological well-being [11–13]. QoL serves as a key metric for evaluating treatment outcomes for IS and is influenced by factors such as the severity of scoliosis, treatment modalities, and the adaptability of individual patients [14,15].

Epidemiologically, the global prevalence of IS is approximately 2–3%, with AIS comprising the majority of cases [16]. AIS typically emerges between the ages of 10 and 18 and exhibits a notable gender disparity: girls not only have a greater incidence but also tend to experience greater curve severity than males do [17,18]. These physical changes, particularly in female patients, often result in heightened psychological distress due to alterations in body image [12, 19]. Consequently, proactive interventions are essential to halt or correct the progression of IS.

Several systematic reviews and meta-analyses have evaluated the effectiveness of exercise-based interventions in managing AIS, particularly in controlling curve progression [20–22].Among these, the Schroth method, spiral stabilization (SPS), and core stabilization exercises have emerged as prominent noninvasive options aimed at alleviating symptoms and potentially correcting spinal curvature [23]. The Schroth method, developed by Katharina Schroth, emphasizes specific exercises tailored to the individual’s curve as well as awareness of posture and breathing techniques that may contribute to improved spinal alignment [24]. This method has shown promise in slowing the advancement of spinal curvature and enhancing the overall quality of life for adolescents diagnosed with IS [25]. In contrast, the SPS approach involves exercises that focus on the dynamic stabilization of the spine and target core muscle engagement to promote balance and strength in the musculature surrounding the spine [26–28]. SPS is predicated on the notion that improved core stability can lead to better postural control and potentially mitigate the effects of scoliosis [23,28]. Core stabilization exercises have been widely implemented as a fundamental aspect of rehabilitation programs for various musculoskeletal conditions, including scoliosis [29,30]. These exercises aim to enhance the function and endurance of the core muscles, thereby supporting the spine and improving overall physical performance [31,32].

The 2016 guidelines of the International Society on Scoliosis Orthopaedic and Rehabilitation Treatment (SOSORT) [33] recommend the following:

Mild curve (Cobb angle 10°–20°): Physiotherapeutic scoliosis-specific exercises (PSSEs) are recommended as the main therapeutic strategy for managing scoliosis.

Moderate curve (Cobb angle of 20°-40°): Bracing is widely recognized as the most effective non-surgical intervention for preventing curve progression, while PSSE are recommended as a valuable adjunct to support brace efficacy and improve patient adherence.

Severe curve (Cobb angle≥40°): Surgical consultation may be needed.

In most cases, adolescent idiopathic scoliosis (AIS) patients with a Cobb angle ≥40° are advised to undergo surgical treatment to prevent further curve progression and improve quality of life. However, recent studies suggest that for patients who refuse surgery, bracing may serve as an effective alternative. A recent systematic review and meta-analysis [34] found that bracing can significantly control the progression of scoliosis even in patients with curves exceeding 40°. These findings offer new insights for clinical decision-making, particularly in cases where patients decline surgical intervention.

Different exercise therapy plans are recommended for PSSE in different countries and regions [33,35]. For example, Schroth therapy was developed in Germany and is widely used there [24]. For mild to moderate curves, particularly in growing patients, targeted physical therapy has shown promise. Compared with patients in general exercise programs, patients who participate in specific scoliosis exercises show significant improvements. These therapies may delay or prevent the need for surgery and reduce the duration or intensity of bracing and are particularly effective for single-curve, low to moderate scoliosis during growth periods [36].

PSSE have been increasingly recognized as a cornerstone of conservative management for AIS, particularly in mild to moderate cases. Among the various approaches, Schroth therapy is the most extensively studied, with evidence supporting its effectiveness in reducing curve progression and delaying the need for bracing during early growth phases [36]. In recent years, alternative methods such as SPS and conventional core stabilization exercises have also gained clinical attention for their potential in improving spinal alignment and neuromuscular control. Several systematic reviews and meta-analyses [20,22,36,37] have suggested that PSSEs are superior to general physiotherapy or no intervention; however, the existing evidence remains limited by small sample sizes, inconsistent methodologies, and short-term follow-ups. Critically, there is a notable lack of studies directly comparing different exercise modalities under the same clinical conditions, making it difficult to draw conclusions about their relative efficacy and practical application. To address this gap, the present retrospective study investigates and compares the outcomes of adolescents with AIS who underwent Schroth therapy, SPS, or traditional core stabilization exercises, aiming to provide clinically relevant evidence that can inform individualized, evidence-based rehabilitation strategies.

## 2. Materials and methods

### 2.1 Study design

This retrospective study included a total of 61 patients with idiopathic scoliosis between June 2021 and June 2024. All subjects were outpatients from the rehabilitation department of Fujian Children’s Hospital and were diagnosed by clinicians with IS. The treatments they received were divided into three groups, the Schroth group, the spiral stabilization (SPS) group and the core stabilization group, for retrospective analysis to determine which treatment was more effective.

This study was conducted in accordance with the ethical principles of the Declaration of Helsinki and approved by the Medical Ethics Committee of Fujian Children’s Hospital (approval number 2024ETKLRK10006) [38]. The requirement for informed consent was waived by the ethics committee due to the retrospective nature of the study based on anonymized data. Throughout the study, the privacy and confidentiality of all participants were carefully maintained. The data were accessed for research purposes in January 2025. Furthermore, all data were anonymized and handled in accordance with applicable data protection regulations [39].

The minimal data set required to replicate the findings of this study has been deposited in Figshare and is available at the following Data Review URL for peer review purposes: https://figshare.com/articles/dataset/scolio_data_xlsx/28424249?file=52381676. A permanent DOI will be provided upon publication.

#### 2.1.1 Sample size calculation.

An a priori power analysis was performed using G*Power software (version 3.1) to determine the required sample size [40]. Based on prior literature, effect sizes for exercise-based interventions in adolescent idiopathic scoliosis (AIS) typically range from medium to large (SMD = 0.6–0.9, approximately corresponding to Cohen’s f = 0.25–0.45) [41–43]. Assuming a significance level of α = 0.05 and a statistical power (1-β) = 0.80, the required sample size for a one-way ANOVA with three groups was estimated to range from 57 to 72 participants. To account for an anticipated dropout rate of 10%, the target sample size was calculated to be between 64 and 80 participants (~21–27 participants per group). Given the retrospective nature of the study, the final sample size was determined by the number of eligible patients available during the study period, resulting in a total of 61 participants. While this is slightly below the upper target range, we consider it sufficiently close to provide meaningful and clinically relevant insights.

### 2.2 Participants

#### 2.2.1 Inclusion criteria.

1. Patients diagnosed with idiopathic scoliosis and a Cobb angle ≤ 40°.
2. Aged 6–18 years.
3. Receiving no additional treatment.
4. Regular outpatient treatment records and detailed pre- and posttreatment evaluations, including X-ray examinations and functional scale assessments

#### 2.2.2 Exclusion criteria.

1. Patients with non-idiopathic scoliosis, congenital malformations or trauma-related complications, and rheumatological or immunological diseases.
2. Patients with previous fractures of the spine/lower limbs or surgical treatment.
3. Psychiatric problems or other contraindications to exercise.

### 2.3 Interventions

The subjects received one of three types of training. For patients with Cobb angles between 20° and 40°, bracing was applied strictly according to the guidelines [33]. Each training was delivered for 30 minutes twice a week, and 10 sessions constituted a course of treatment. All included subjects participated in at least one course of treatment. All training sessions were supervised by a professional physical therapist, and children under 12 years of age were additionally supervised by their parents. Patients who received training in the hospital were assigned homework with the same frequency and time so that they could continue to follow the training.

#### 2.3.1 Schroth group.

**Fig 1:**
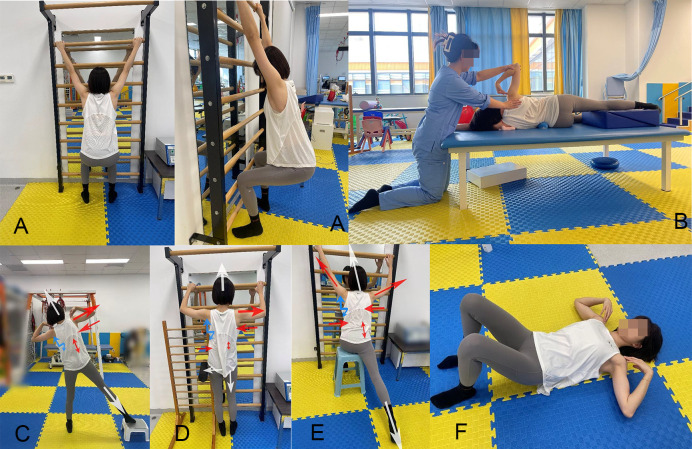
Schroth group programs. **(A)** Great arch; **(B)** Side-Lying muscle cylinder exercise; **(C)** Standing muscle cylinder exercise; **(D)** Standing hip push on a stall bar; **(E)** Rotational sitting exercise; **(F)** Supine breathing correction (see detailed exercise descriptions in Supplementary File S1 File).

Each session lasted 30 minutes and focused on Schroth exercises specifically tailored to the patient’s type of scoliosis. The program was guided by a trained physiotherapist and emphasized precise execution, individualized adjustments, and continuous progress monitoring to ensure optimal outcomes. Each exercise targeted spinal alignment correction, self-elongation, derotation with corrective breathing and muscular balance and was typically performed for 5–10 minutes per movement. The training included targeted movements such as the “great arch,” which involves spinal elongation and thoracic expansion, and the “side-lying muscle cylinder exercise,” which focuses on breathing into the concave areas while maintaining a neutral spine alignment. Other exercises, such as “rotational sitting,” address pelvic and spinal alignment and precise postural adjustments, whereas “standing muscle cylinder exercise” and “supine breathing correction” enhance spinal elongation, respiratory mechanics, and muscular balance through structured movements and controlled breathing (see Fig 1). Breathing correction aims to establish intra-abdominal pressure and promote spinal alignment [44–46]. Detailed information on the exercises is presented in S1 File.

#### 2.3.2 Spiral stabilization (SPS) group.

**Fig 2:**
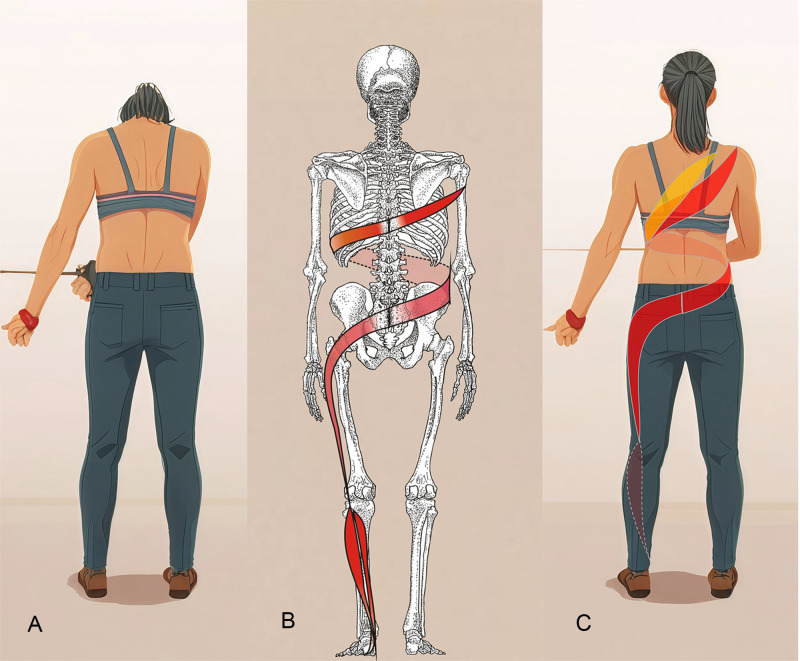
SPS exercise (Central Muscle Band Stabilization Movements). **(A)** Start position; **(B)** Activated muscle chains; **(C)** End position (see detailed exercise descriptions in Supplementary File S2 File).

**Fig 3:**
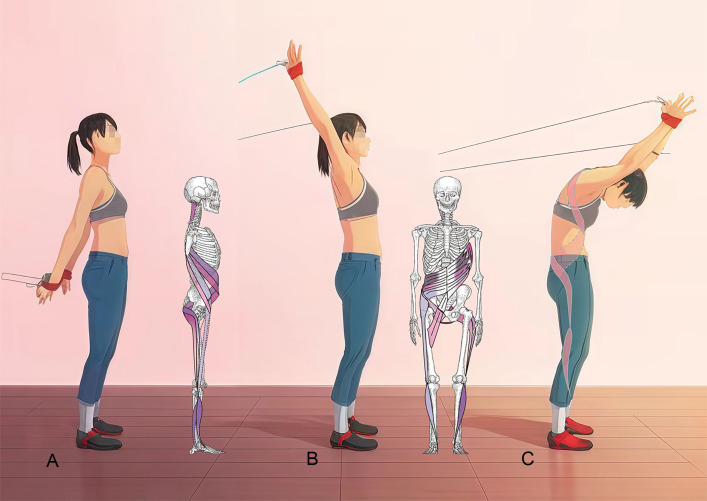
SPS exercise (Peripheral Muscle Band Coordination). **(A)** Start position; **(B)** Transition position; **(C)** End position(see detailed exercise descriptions in Supplementary File S2 File).

Each session consisted of 30 minutes of SPS exercises guided by a trained physiotherapist. The program emphasized precise execution, personalized adjustments, and continuous monitoring to ensure optimal rehabilitation outcomes. Each exercise is intended to correct spinal alignment, activate spiral muscle chains, and enhance balance and mobility. Movements typically last 5–10 minutes with a focus on spinal elongation, core stabilization, and muscle coordination. Key exercises include “central plane alignment,” which targets spinal straightening and restoring natural curvatures, and “gait optimization,” which integrates stability and symmetry through walking drills. Other targeted practices, such as “peripheral muscle band coordination,” enhance shoulder and pelvic girdle flexibility, whereas “central muscle band stabilization movements” are intended to improve strength and functional movement patterns (see Figs 2 and 3). All exercises adhered to the SPS principles of vertical axis training and emphasized muscle balance, symmetrical movement, and gait refinement [26]. SPS exercise is highly personalized and is based on the patient’s ability and training principles, which are outlined in S2 File.

#### 2.3.3 Core stabilization group.

**Fig 4:**
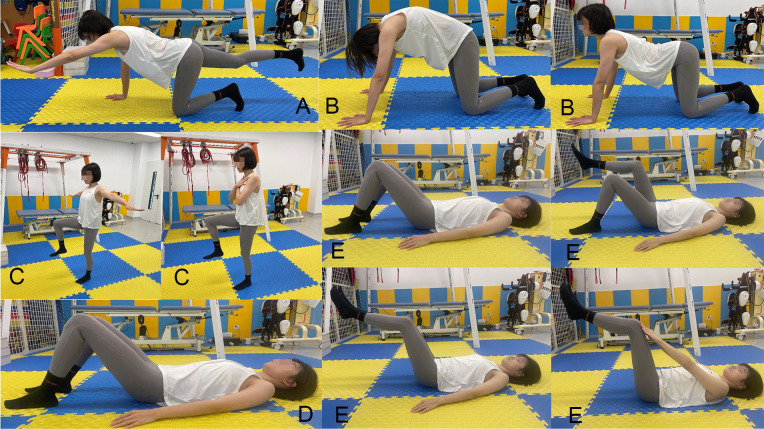
Core stabilization group programs. **(A)** All-fours exercise; **(B)** Cat-camel exercise; **(C)** Single leg balance; **(D)** Pelvic tilts; **(E)** Double-leg abdominal press(see detailed exercise descriptions in Supplementary File S3 File).

Each session consisted of 30 minutes of core stabilization exercises guided by experienced physiotherapists. Key movements include the “all fours exercise,” which involves controlling arm and leg extensions to activate glutes and stabilize the core, and the “cat-camel exercise,” which focuses on spinal mobility and breathing coordination. Other exercises, such as “single leg balance,” improve stability and coordination, whereas “pelvic tilts” and “double-leg abdominal press” engage the abdominal muscles to increase lumbar support and core strength [29,47] (see Fig 4). All exercises were structured to progressively build balance and maintain a neutral spinal alignment. The detailed program of the exercises is presented in S3 File.

### 2.4 Outcome measurements

The basic data of the subjects, including age, sex, and body mass index (BMI), were collected before the first outpatient treatment. Other parameters included pain (VAS scale) [48,49], angle of trunk rotation (ATR) [50,51], full-length spine radiographs, trunk aesthetic clinical evaluation (TRACE) [52,53], and the Scoliosis Research Society-22 questionnaire (SRS-22) [54,55]. These data were collected again at a follow-up visit 6 months later. All data were collected and organized by the same researcher. The full-length spine radiographs were measured to obtain the corresponding Cobb angle, Risser sign, and alignment of the C7 plumbline (C7PL) in relation to the central sacral vertical line (CSVL) (C7PL-CSVL) [56–58], clavicle angle, and pelvic obliquity. In this study, all radiographic measurements were performed by the same experienced physical therapist, who was blinded to the patients’ treatment group assignments in order to minimize potential measurement bias. This study examined the degree of improvement of the subjects in different groups after treatment by combining the analysis of imaging indicators with functional assessment scales.

#### 2.4.1 Primary outcome.

The Cobb angle is measured by full-length radiographs of the spine. This method was invented by Dr. John Cobb in 1948 [59]. The Cobb angle is widely recognized as the key measurement for evaluating and tracking the progression of scoliosis. It is determined by measuring the curvature angle by first drawing lines parallel to the upper edge of the highest vertebral body and the lower edge of the lowest vertebral body within the affected curve. After these reference lines are established, perpendicular lines are drawn from them that meet at a point where the curvature angle is determined. This measurement provides a precise and reliable metric for assessing spinal deformities while ensuring consistency in the clinical diagnosis and longitudinal monitoring of scoliosis progression [60,61]. The Cobb angle referred to in this study is the largest angle of the bend measured in the patient’s imaging.

#### 2.4.2 Secondary outcomes.

**Visual Analog Scale (VAS):** The visual analog scale (VAS) is a pain assessment tool invented by Hayes and Patterson in 1921. It is widely used in epidemiological and clinical research. This method assesses pain on a continuous range from none to extreme. Unlike the classification scale, which divides pain into categories such as none, mild, moderate, and severe, the VAS reflects a continuous view of pain without a clear division. The VAS usually has a 10-centimeter straight line with end marked “no pain” and the other end marked “worst pain.” Patients mark a point on the line to indicate their perceived level of pain. By measuring this point, a value representing the intensity of pain can be obtained. This design provides a more accurate and subjective measurement of pain and is in line with the continuity of the pain experience perceived by patients.

**The Scoliosis Research Society-22 questionnaire (SRS-22):** The SRS-22 is a tool specifically designed to evaluate health-related quality of life (HRQoL) across five key dimensions. It consists of 22 items, with five questions dedicated to each of four categories (i.e., function, pain, self-image, and mental health) along with two items that assess treatment satisfaction. Respondents rate each question on a scale ranging from 1 to 5 points. The Chinese version of the SRS-22 questionnaire used in this study has demonstrated strong reliability and validity, confirming its effectiveness as a suitable instrument for assessing HRQoL among patients with adolescent idiopathic scoliosis in mainland China [62,63].

**Angle of trunk rotation (ATR):** The assessment of trunk rotation is conducted using a scoliometer while the patient is in the Adam’s forward flexion test position. In this stance, the individual bends forward at the waist until the back is horizontal with the feet together, arms hanging freely, and knees straight. Once the patient is in this position, the scoliometer is placed vertically along the spine at the location of the rib hump. The segment with the greatest inclination angle is identified, and the corresponding angle of trunk rotation (ATR) is recorded [64–66].

**Trunk aesthetic clinical evaluation (TRACE):** Aesthetic enhancement is crucial in evaluating treatment outcomes for adolescent idiopathic scoliosis (AIS), yet there are currently no established standards for this assessment. The TRACE tool was developed to measure symmetry and serves as an indicator of aesthetic quality in patients. This assessment consists of four key components: shoulder (scored from 0 to 3), scapula (0–2), hemi-thorax (0–2), and waist (0–4). The overall score is calculated by summing these four components and adding an additional point, resulting in a total score that ranges from 1 to 12. TRACE has shown adequate reproducibility, making it a suitable option for incorporation into standard clinical practice [52,53].

**Other radiographic outcomes:** In addition to the Cobb angle, full-length anteroposterior spine radiographs can reveal improvements in coronal balance in patients with IS. All data were processed and acquired by the same researcher utilizing Image-Pro Plus software (version 7.0; Media Cybernetics, Inc., USA). All image data were measured and analyzed in a blinded manner. To ensure data quality, all measurements were performed twice, and the final data were obtained by averaging the two results.

1. Alignment of the C7 plumbline (C7PL) in relation to the central sacral vertical line (CSVL) (C7PL-CSVL)

A plumb line should be established at the midpoint of the C7 vertebra, known as the C7 plumb line (C7PL). In a similar manner, a plumb line should be drawn at the midpoint of the S1 vertebra on the X-ray, which is referred to as the central sacral vertical line (CSVL) [56–58,67], and the horizontal distance between the two plumb lines should be measured, which is considered the offset distance. Theoretically, these two straight lines overlap for people without scoliosis.

2. Clavicle angle

The clavicle angle is the angle between the line connecting the highest points of the clavicles at both ends (conoid tubercle) on the X-ray and the horizontal reference line (HRL) [67,68].

3. Pelvic obliquity

This study introduces the pelvic coronal reference line (PCRL), which is created by connecting the tips of the sacral ala on both sides. This method offers a more precise depiction of the coronal deviation of the pelvis. The angle formed between the PCRL and the horizontal reference line (HRL) is used to indicate the pelvic inclination angle [68,69].

### 2.5 Statistical analysis

Statistical analysis of the data was conducted using SPSS® software version 25.0 (IBM® Corp., Armonk, NY, USA). The Shapiro-Wilk test was used to evaluate the normality of distribution, and Levene’s test was used to test the equality of variance. Data was presented as mean ± standard deviation for normally distributed variables and median (interquartile range) for non-normally distributed variables. For within-group comparisons (pre- and posttreatment), paired t tests were conducted. Between-group differences were assessed using the chi-square test for categorical variables, one-way analysis of variance for normally distributed continuous variables followed by the least significant difference (LSD) post hoc test for pairwise comparisons, and the nonparametric Kruskal-Wallis test was used for non-normally distributed variables. A significance level of p < 0.05 was set for all analyses.

## 3. Results

### 3.1 Baseline characteristics

In this study, a total of 61 patients were enrolled and divided into three distinct groups: the Schroth group, which included 22 patients; the spiral stabilization (SPS) group, which included 21 patients; and the core stabilization group, which included 18 patients. An analysis of the baseline characteristics revealed no statistically significant differences among the three groups in terms of gender distribution (p = 0.064), age (p = 0.055), body mass index (BMI) (p = 0.121), Risser sign (p = 0.732), or Cobb angle distribution (10°–20° vs. 20°–40°; p = 0.386), indicating that the potential confounding effect of bracing was relatively balanced across interventions (Table 1).

**Table 1:** Demographic characteristics of the participants by group and p values for group differences.

	Schroth (n = 22)	SPS (n = 21)	Core (n = 18)	P
Gender				0.064
Male	3 (13.64%)	7 (33.33%)	1 (5.56%)	
Female	19 (86.36%)	14 (66.67%)	17 (94.44%)	
Age (years)	10.73 (2.25)	12.14 (1.59)	11.50 (1.69)	0.055
BMI (kg/m2)	21.35 (2.62)	20.26 (2.02)	19.93 (2.13)	0.121
Cobb 10–20°(no brace)	13 (59.09%)	12 (57.14%)	7 (38.89%)	0.386
Risser sign				0.732
0	13 (59.09%)	9 (42.86%)	11 (61.11%)	
1	4 (18.18%)	2 (9.52%)	2 (11.11%)	
2	2 (9.09%)	2 (9.52%)	2 (11.11%)	
3	1 (4.55%)	2 (9.52%)	2 (11.11%)	
4	2 (9.09%)	5 (23.81%)	1 (5.56%)	
5	0 (0.0%)	1 (4.76%)	0 (0.0%)	

Continuous variables are expressed as the mean ± standard deviation (SD).

Categorical variables were analyzed via the chi-square test and are expressed as numbers and percentages.

### 3.2 Imaging and functional outcomes

#### 3.2.1 Within-group comparisons.

The results of all three groups revealed significant differences (p < 0.05) in some indicators (Table 2).

**Table 2:** Intra- and intergroup comparisons of the Cobb angle, VAS score, ATR, C7PL alignment, clavicle angle, pelvic angle, and TRACE score.

	Schroth		SPS		Core		Schroth vs. SPS	Schroth vs. Core	SPS vs. Core
	Primary	6 months	Primary	6 months	Primary	6 months			
Cobb(°)	20.32 (8.62)	11.00 (7.40)*	20.52 (8.66)	11.71 (9.83)*	24.44 (8.48)	17.72 (8.22)*	0.785	0.016*	0.033*
VAS	1.23 (1.57)	0.23 (0.43)*	1.71 (1.52)	0.57 (1.03)*	1.56 (1.62)	0.39 (0.85)*	0.165	0.528	0.481
ATR(°)	7.55 (2.79)	4.14 (2.40)*	8.57 (3.17)	5.00 (3.11)*	11.28 (4.65)	8.00 (4.59)*	0.409	0.001*	0.008*
C7PL(cm)	1.28 (1.06)	0.59 (0.68)*	0.96 (1.12)	0.62 (0.64)	1.62 (1.21)	1.07 (0.70)	0.911	0.030*	0.041*
Clavicle Angle(°)	2.04 (1.87)	1.43 (1.24)	2.41 (1.25)	0.50 (0.22)*	2.72 (1.82)	1.83 (1.37)	0.006*	0.240	<0.001*
Pelvic angle(°)	1.88 (1.00)	0.91 (0.90)*	1.86 (1.42)	0.32 (0.33)*	1.62 (1.02)	1.39 (0.85)	0.011*	0.047*	<0.001*
TRACE	4.50 (2.39)	2.73 (2.75)*	4.67 (1.59)	1.95 (1.47)*	7.22 (2.10)	4.89 (1.91)*	0.238	0.002*	<0.001*

* indicates statistical significance at a p value < 0.05.

Continuous variables are presented as the mean values along with their standard deviations (SDs).

In the Schroth group, the Cobb angle decreased from 20.32 ± 8.62° to 11.00 ± 7.40° (p < 0.05), representing a statistically significant improvement (p < 0.05). The SPS group exhibited a statistically significant decrease from 20.52 ± 8.66° to 11.71 ± 9.83° (p < 0.05). The core stabilization group also showed a statistically significant reduction from 24.44 ± 8.48° to 17.72 ± 8.22° (p < 0.05).

In the Schroth group, the VAS score improved from significantly 1.23 ± 1.57 to 0.23 ± 0.43 (p < 0.05). The SPS group also demonstrated a statistically significant reduction in VAS scores from 1.71 ± 1.52 to 0.57 ± 1.03 (p < 0.05), while the core stabilization group showed a statistically significant reduction from 1.56 ± 1.62 to 0.39 ± 0.85 (p < 0.05).

The Schroth group exhibited a statistically significant improvement in ATR, decreasing from 7.55 ± 2.79° to 4.14 ± 2.40° (p < 0.05). The SPS group also showed a statistically significant improvement from 8.57 ± 3.17° to 5.00 ± 3.11° (p < 0.05), as did the core stabilization group from 11.28 ± 4.65° to 8.00 ± 4.59° (p < 0.05).Regarding the alignment of the C7 plumbline (C7PL-CSVL), the Schroth group demonstrated a statistically significant improvement from 1.28 ± 1.06 cm to 0.59 ± 0.68 cm (p < 0.05). The SPS group showed a non-significant reduction from 0.96 ± 1.12 cm to 0.62 ± 0.64 cm (p > 0.05). Similarly, the core stabilization group did not show a statistically significant change from 1.62 ± 1.21 cm to 1.07 ± 0.70 cm (p > 0.05).

In terms of the clavicle angle, the SPS group demonstrated a statistically significant improvement from 2.41 ± 1.25° to 0.50 ± 0.22° (p < 0.05). The Schroth group exhibited a non-significant reduction from 2.04 ± 1.87° to 1.43 ± 1.24° (p > 0.05), as did the core stabilization group from 2.72 ± 1.82° to 1.83 ± 1.37° (p > 0.05).

For the pelvic angle, the Schroth group showed a statistically significant improvement from 1.88 ± 1.00° to 0.91 ± 0.90° (p < 0.05), as did the SPS group from 1.86 ± 1.42° to 0.32 ± 0.33° (p < 0.05). The core stabilization group exhibited a non-significant reduction from 1.62 ± 1.02° to 1.39 ± 0.85° (p > 0.05).

The TRACE scores were significantly reduced in all groups: in the Schroth group from 4.50 ± 2.39 to 2.73 ± 2.75 (p < 0.05), in the SPS group from 4.67 ± 1.59 to 1.95 ± 1.47 (p < 0.05), and in the core stabilization group from 7.22 ± 2.10 to 4.89 ± 1.91 (p < 0.05).

#### 3.2.2 Between-group comparisons.

The between-group analyses demonstrated notable variations in outcomes among the three groups (Table 2). Compared with the core stabilization group, the Schroth group exhibited a significantly greater reduction in the Cobb angle (p = 0.016), and the SPS group also showed a significantly greater reduction (p = 0.033). No significant difference was observed between the Schroth and SPS groups (p = 0.785).No statistically significant differences were detected regarding improvement in VAS scores among the three groups. The decrease in VAS scores was comparable between the Schroth and SPS groups (p = 0.165), the Schroth and core stabilization groups (p = 0.528), and the SPS and core stabilization groups (p = 0.481).

For ATR, the Schroth group exhibited a significantly greater reduction compared to the core stabilization group (p = 0.001), as did the SPS group (p = 0.008). No significant difference was found between the Schroth and SPS groups (p = 0.409).Regarding C7PL alignment, the Schroth group demonstrated a significantly greater improvement compared to the core stabilization group (p = 0.030), as did the SPS group (p = 0.041). No significant difference was observed between the Schroth and SPS groups (p = 0.911).

In terms of the clavicle angle, the Schroth group demonstrated a significantly greater improvement compared to the core stabilization group (p < 0.001), and the Schroth group demonstrated a significantly greater improvement compared to the SPS group (p = 0.006). The comparison between the Schroth group and the core stabilization group did not reveal a statistically significant difference (p = 0.240).

For the pelvic angle, the Schroth group demonstrated a significantly greater reduction compared to both the SPS group (p = 0.011) and the core stabilization group (p = 0.047). The SPS group also demonstrated a significantly greater reduction compared to the core stabilization group (p < 0.001).Finally, compared with the core stabilization group, both the SPS group (p < 0.001) and the Schroth group (p = 0.002) demonstrated significantly greater reductions in the TRACE score. No significant difference was observed between the Schroth and SPS groups (p = 0.238).

#### 3.2.3 Quality of life (SRS-22).

All three groups showed no significant between-group differences across all dimensions of the SRS-22, including pain, self-image, function, mental health, and satisfaction (p > 0.05 for all).

In the within-group comparisons, all three treatments (Schroth, SPS, and core stabilization) demonstrated statistically significant improvements in pain, self-image, and mental health (p < 0.05 for all). For functional scores, a statistically significant improvement was found only within the SPS group (p < 0.05), whereas the Schroth and core stabilization groups showed no significant changes (p > 0.05).

Because patients assessed their satisfaction only after treatment, the statistical significance of the changes in this domain could not be evaluated due to the lack of baseline data (Table 3).

**Table 3:** Intra- and Intergroup Comparisons of Quality of Life (SRS-22).

	Schroth		SPS		Core		Schroth vs. SPS	Schroth vs. Core	SPS vs. Core
	Primary	6 months	Primary	6 months	Primary	6 months			
Pain	2.01 (0.25)	1.84 (0.19)*	2.08 (0.25)	1.88 (0.15)*	2.12 (0.32)	1.87 (0.22)*	All *P* > 0.05		
Self-image	2.71 (0.89)	1.96 (0.71)*	2.98 (0.92)	1.86 (0.70)*	2.57 (0.86)	1.80 (0.76)*			
Function	2.90 (0.25)	2.86 (0.22)	3.10 (0.43)	2.94 (0.27)*	2.91 (0.32)	2.89 (0.30)			
Mental health	2.73 (0.21)	2.55 (0.23)*	2.78 (0.28)	2.54 (0.22)*	2.80 (0.24)	2.62 (0.17)*			
Satisfaction	–	1.43 (0.54)	–	1.60 (0.85)	–	1.56 (0.62)			

* indicates statistical significance at a p value < 0.05.

Continuous variables are presented as the mean values along with their standard deviations (SDs).

## 4. Discussion

The analysis of three approaches to physical therapy (Schroth, spiral stabilization (SPS), and core stability exercises) highlights their respective advantages and limitations in treating idiopathic scoliosis. All three interventions effectively reduce the Cobb angle and trunk rotation angle (ATR) and improve pain (as measured by the visual analog scale (VAS)).

In interpreting the radiographic findings, it is important to distinguish between statistical and clinical significance. Although our study demonstrated statistically significant reductions in Cobb angle within all three groups, it is the magnitude of these changes that strengthens their clinical relevance. Specifically, all groups demonstrated mean Cobb angle reductions exceeding 5°, the conventional threshold used to account for inter-rater and intra-rater measurement variability [33]. This suggests that the improvements observed likely reflect genuine structural changes rather than measurement artifacts. Future studies incorporating MCID-based responder analysis at the individual level would further strengthen the interpretability of these findings.

However, core stability exercises demonstrate lower specificity in addressing scoliosis-related issues. While core exercises can promote muscle balance and endurance, they lack the corrective focus on spinal neutral alignment emphasized by the Schroth and SPS methods [70,71].The improvement in the pelvic tilt in the core stability group was negligible because these exercises prioritize muscle symmetry rather than directly controlling pelvic alignment. This contrasts sharply with the Schroth and SPS therapies, which combine strategies to address coronal and sagittal imbalances. Schroth emphasizes respiratory mechanics and rib correction, whereas SPS focuses on coordinating pelvic and scapular stability [44,45]. This distinction makes Schroth more suitable for addressing three-dimensional deformities, although it may sometimes overcorrect thoracic curves, especially in types 3B/3BH [26,70].

With respect to improvements in the clavicular angle, SPS achieved the greatest progress due to its emphasis on dynamic postural control and scapular balance. In contrast, Schroth targets rib opening mechanics through three-dimensional derotation breathing but often neglects compensation of the scapula and clavicle. The static nature of Schroth training limits its dynamic functional carryover, whereas SPS enhances dynamic movement control, which may benefit daily activities and gait [26,28]. These findings suggest that future research should incorporate dynamic assessment tools such as electromyography to better evaluate functional improvements [29].

For C7 plumb line alignment (C7PL), only Schroth showed significant improvement. This result may stem from Schroth’s focus on body awareness and static postural correction, which aids in achieving a neutral position of the spine. However, given that humans walk thousands of steps daily, the lack of dynamic movement training in Schroth may limit its impact on daily activities. SPS addresses this gap by targeting dynamic postural adjustments, which highlights its potential to integrate scoliosis treatment into functional tasks [49,70].

According to the SRS-22 assessment, all groups may have experienced improvements in certain aspects of quality of life; however, no statistically significant differences were observed between groups [42,72]. This result should be interpreted with caution, as potential ceiling effects and the limited sensitivity of the scale may have influenced the ability to detect subtle changes. Nevertheless, participation in structured physical therapy programs may contribute to enhanced psychological well-being and perceived physical function [73]. Among the three treatments, SPS may offer additional benefits in functional outcomes, potentially due to its emphasis on dynamic movements such as gait optimization, which support patients’ reintegration into social and family activities. Furthermore, active engagement in scoliosis treatment is known to foster psychosocial involvement [74], which may complement the physical effects of therapy, although further research with more sensitive assessment tools is needed to substantiate this impact.

Although all radiographic measurements in this study were performed by the same experienced rehabilitation therapist who was blinded to treatment group assignments, some potential residual sources of measurement bias remain. Intra-rater variability is inherent in manual Cobb angle measurements, and small measurement errors have been documented even under blinded and standardized conditions. Additionally, variability in patient positioning during standing radiographs may introduce minor inconsistencies in spinal alignment visualization. These limitations should be considered when interpreting the radiographic outcomes of this study.

The relatively short duration of the intervention and follow-up (6 months) limits our ability to fully assess the long-term effects and sustainability of treatment outcomes [25,75]. This is particularly relevant given that the participants’ mean age was approximately 10 years, placing them at the early stage of the peak growth phase, during which scoliosis is most likely to progress. We fully acknowledge the importance of extended follow-up—ideally continuing through skeletal maturity—for accurately capturing the natural course of curve progression and the long-term effects of treatment. Moreover, the wide age range of included patients (6–18 years) may introduce heterogeneity in both treatment compliance and physiological response to therapy, as juvenile and adolescents often difference in terms of skeletal maturity, neuromuscular development, and motor skill proficiency. Similarly, early-onset scoliosis may differ pathophysiologically from later-onset cases, potentially influencing responsiveness to conservative interventions [76]. While the Risser sign was recorded as a marker of skeletal maturity and discussed accordingly, further stratification or subgroup analysis based on age and onset type may be valuable in future studies [77,78].In accordance with the SOSORT guidelines, we recommend follow-up examinations for patients after 6 months [33]. However, patients may face social and familial challenges, such as heavy academic burdens, which prevent them from coming to the hospital for follow-up. This may lead to slight inconsistencies in the timing of these assessments and potentially bias the results. Similarly, differences in patients’ self-discipline and compliance may lead to inconsistencies in the intensity and frequency of home training that cannot be monitored [79]. This may result in variations in follow-up outcomes.

Another limitation of this study is the lack of quantitative objective data, which limits the comprehensiveness assessment of the results of physical therapy outcomes [80]. Specifically, reliance on subjective measures such as the SRS-22 questionnaire and TRACE scores, and VAS introduces a degree of subjectivity, as these outcomes are inherently influenced by patients’ perceptions and inter-observer variability [81]. While patient-reported outcomes remain essential for capturing the patient’s perspective on functional and quality-of-life improvements, as emphasized by current SOSORT guidelines, they alone are insufficient to comprehensively reflect the biomechanical and physiological adaptations elicited by treatment. Objective biomechanical assessments, such as surface electromyography (sEMG) [82,83], gait analysis, and isokinetic dynamometry, can provide valuable complementary insights into muscle activation patterns, motor control, and functional performance. Accordingly, we have explicitly addressed this point to underscore the need for future studies to integrate both patient-reported and objective biomechanical measures in order to achieve a more comprehensive evaluation of treatment effects and to deepen our understanding of the underlying mechanisms driving therapeutic improvements.

Bracing is an important confounding factor in scoliosis management, as guidelines recommend its use when the Cobb angle exceeds 20° but not below this threshold [33]. Although some studies suggest that bracing may enhance the effects of exercise therapy [84], our analysis showed that the distribution of patients in the 10°–20° versus 20°–40° categories was relatively balanced across groups (Schroth: 59%, SPS: 57%, Core stabilization: 39%), with no significant differences observed (p = 0.386, p > 0.05; Table 1). This indicates that bracing is unlikely to account for the differences in outcomes. While excluding younger patients or those using braces might reduce potential bias, it would also substantially lower statistical power and limit the ability to detect meaningful effects. We therefore retained these participants and acknowledge this as a limitation.

Additionally, the Risser sign has not been optimally managed, which may lead to inconsistencies in the potential growth of the patient population [85]. This is a double-edged sword: a smaller Risser sign indicates a greater risk of disease progression for the patient but also suggests a greater possibility of recovery, thereby affecting the consistency of treatment responses [86]. Early detection and early treatment are important. Future research should control these variables to gain a clearer understanding of the optimal timing of interventions for IS [87].

The relatively small sample size of this study—particularly in the core stabilization group—limits the statistical power to detect potentially meaningful between-group differences, especially for secondary outcomes such as SRS-22 quality of life and VAS pain scores. The non-significant findings observed for these subjective outcomes should therefore be interpreted with caution, as they may reflect insufficient statistical power rather than a true absence of effect. This limitation hindered our ability to determine whether certain physical therapy regimens were more beneficial for specific subtypes of scoliosis [88,89].

The non-randomized, retrospective design inherently introduces selection bias, limiting the ability to establish causal relationships. In particular, a baseline imbalance was observed in Cobb angle between groups, with the core stabilization group presenting with a higher mean baseline value, which may have influenced the comparability of treatment outcomes. Second, important confounding factors such as home exercise compliance and socioeconomic status were not fully controlled, although standardized exercise instructions and routine clinical monitoring were implemented [90]. Residual confounding cannot be ruled out and may have affected the results. Future randomized, prospective studies with rigorous confounder control will be essential to more definitively evaluate the efficacy of different exercise interventions in AIS and to clarify their causal impact on clinical outcomes.

This study focused primarily on coronal imaging assessments, utilizing the Cobb angle and related anteroposterior radiographic parameters, without incorporating sagittal imaging for a comprehensive evaluation of spinal alignment [91]. An important consideration in this design was the need to minimize radiation exposure in adolescent patients [92,93], which constrained subsequent imaging assessments to standing full-length anteroposterior radiographs. As a result, this study did not assess potential changes in sagittal alignment, including thoracic kyphosis, lumbar lordosis, and pelvic parameters, which are critical for a comprehensive understanding of spinal deformities and treatment responses [91], such as spinal kyphosis, spinal lordosis, and pelvic rotation, which are crucial for a comprehensive understanding of spinal deformities. Future research should consider the use of advanced low-radiation imaging technologies, such as three-dimensional spinal assessment systems, to evaluate the three-dimensional characteristics of scoliosis, including spinal and pelvic deformities [94,95]. These methods can enhance the accuracy and comprehensiveness of scoliosis assessments and provide a more detailed understanding of treatment outcomes [96].

This study lacked an assessment of spinal flexibility, which may impact the treatment results [97]. Previous research has revealed a negative correlation between the Cobb angle, spinal rotation, and seated forward bending test results in patients with AIS, indicating that a larger Cobb angle and greater degree of spinal rotation are associated with poorer lumbar flexibility [98]. Improving spinal flexibility and mobility is crucial for the conservative treatment of scoliosis [96,97]. The lack of flexibility assessment in this study overlooks a key dimension in the understanding of the relationship between flexibility improvement and treatment outcomes. This omission limits the ability to comprehensively evaluate the therapeutic benefits of training programs and their impact on correcting spinal deformities and potentially reduces the generalizability of the findings. Future research should address this gap by incorporating flexibility assessments to elucidate the multifaceted effects of physical therapy interventions on scoliosis correction and functional outcomes.

Moreover, as emphasized in the SOSORT guidelines [33], current evidence regarding the efficacy of conservative treatment is strongest for adolescents with moderate scoliosis (Cobb angle ≤40°). Extrapolation of our findings to adults or patients with more severe curves should be made with caution, given the limited evidence base and the differing biomechanical and clinical profiles of these populations. In addition, future research should focus specifically on patients aged 10–18 years with Cobb angles between 10° and 20°, for whom exercise serves as the primary intervention, as this may provide more definitive evidence regarding treatment efficacy. Despite the promising findings, several limitations should be acknowledged. The retrospective design, relatively small sample size, and short follow-up period may affect the robustness and generalizability of the results. A major limitation of this study is that follow-up was confined to the active treatment period, which does not satisfy the SRS criterion for defining long-term success—maintenance of the final Cobb angle within ±5° of baseline two years after the cessation of non-surgical therapy. As a result, the outcomes reported here capture only short-term effects. Future prospective studies with extended follow-up, ideally continuing for at least two years after treatment completion, are needed to establish the durability and long-term efficacy of these exercise-based interventions [33].

## 5. Conclusion

This short-term retrospective analysis demonstrated that Schroth, SPS, and core stabilization exercises all improved Cobb angle and ATR in patients with AIS, although core stabilization was slightly less effective. Schroth and SPS offered additional aesthetic benefits, but all three had limited effects on pain and quality of life. These findings support their use as conservative, noninvasive treatments for AIS.

## Supporting information

S1 File Schroth group programs. (DOCX)

S2 File SPS group programs. (DOCX)

S3 File Core stabilization group programs. (DOCX)
