# Supplementary material for: Effects of different physical therapy training protocols on patients with idiopathic scoliosis: Short-term results
Source: PLoS One. 2025 Oct 17;20(10):e0334713. doi: 10.1371/journal.pone.0334713 (PMC12533888; doi:10.1371/journal.pone.0334713)
Supplement: S1 File — (DOCX) [file pone.0334713.s001.docx]

**Schroth group programs**

The total training time is 30 minutes, and in principle each movement takes 5 minutes，and the specific movement is selected or fine-tuned according to the type of scoliosis.

1. Great arch

First, place your hands on the highest reachable bar of the stall bars, then move to a lower bar.

- Slightly bend your knees.

- Push your hips back and extend your spine.

- Open the narrowed front chest/expand the armpit and ribs on the side with the rib protrusion.

- Adjust to a neutral position.

Perform the breathing framework:

1. Inhale: Extend fully.

2. Exhale quickly.

3. Inhale: Focus on expanding the concave side.

4. Exhale: Relax your shoulders.

2. Side-Lying Muscle Cylinder Exercise

Side-Lying Breathing Position: Initial Setup

- Lie on your side with the concave side of the lumbar spine (or the convex side of the thoracic spine) facing upward.

- Place the upper hand on the shoulder apex, fingers gripping firmly.

Breathing Instructions:

- Inhale: Direct the breath into the concave areas of the thoracic and lumbar spine.

- Exhale: Close the convex side while relaxing and lowering the shoulders.

- Extend the lower limbs away from the body.

Lower Limb Guidance:

- Keep the lower limbs slightly off the support surface, maintaining a neutral position.

- Optional: Add weights or resistance for increased difficulty.

- Note:The lower limb movement is not recommended for patients without lumbar scoliosis.

3. Rotational Sitting Exercise

- Sit on a chair. Stretch and externally rotate the hip on the concave side. Push the pelvis downward and backward.

- Place the other leg in front, bent at a 90° angle, keeping the upper body aligned with the front leg. Shift your body weight toward the pelvis on the concave side.

- Place a correction wedge under the ischium on the concave side to adjust pelvic rotation (applicable for patients with pelvic obliquity correction needs).

- Tilt the pelvis downward on the concave side and forward, but avoid overly emphasizing concave rotation (to strengthen the inactive muscles on the concave side).

- Shift the head in the opposite direction (toward the convex side of the spine). Slightly tilt the head toward the concave side to activate the deep weak muscles of the concave side, which provide support for movement correction. Avoid excessive neck tension.

- Ensure the lumbar spine remains aligned with the midline. During exercise, the lumbar spine should gradually approach the midline, activating the muscles on the concave side of the waist.

4. Standing Muscle Cylinder Exercise

Preparation Position

1. Step or Bench Setup:

- Find a step or bench of appropriate height and place it on the ground.

2. Standing Posture:

- Maintain a neutral standing position.

- Place the leg on the concave side of your lumbar spine on the step, ensuring it provides stable support on the ground.

- Keep your pelvis stable and aligned.

- Shift most of your body weight onto the leg on the convex side of the lumbar spine.

Arm and Torso Adjustment

1. Hand Position:

- Bend your arms and place your hands on your shoulder tips.

- Open your elbows outward to help expand your chest and promote shoulder stability and flexibility.

2. Torso Side-Bending:

- Slowly bend your upper body slightly toward the concave side of the thoracic spine, as if your leg is moving away from the step.

- This movement aims to correct spinal curvature and stretch the muscles on the concave side of your body.

Spinal Extension

1. Head Position:

- Imagine a string pulling the top of your head upward, keeping your spine as extended as possible.

- Push your head upward, elongating your spine as if resisting gravity to lengthen it further.

Leg Movement

1. Leg on the Step:

- Keep the leg on the step stable, and gently push your foot outward.

- This outward push helps maintain pelvic stability and counter spinal rotation.

2. Supporting Leg:

- Ensure the supporting leg remains slightly bent to avoid locking the knee and reduce pressure on the joint.

Breathing Exercise

1. Inhale:

- Direct the breath into the concave side of your thoracic and lumbar spine.

- Visualize your breath expanding your chest cavity, opening the concave areas as much as possible.

2. Exhale:

- Slowly release the breath, allowing the convex side of your spine to gently close.

5. Supine Breathing Correction (Starting from a Supported Position)

Step 1: Adjust to a Neutral Position

1. Back Position:

- Flat back: Press palms downward into the floor.

- Rounded back (kyphosis): Turn palms upward.

2. Shoulder Compensation:

- If shoulder shrugging occurs:

- Flat back: Hold a yoga ball in front of you.

- Rounded back: Place shoulders in 90° abduction and 90° elbow flexion.

3. Neck:

- Use a yoga block to guide the neck closer to a neutral position or provide gentle support to limit excessive movement.

4. Pelvis:

- Correct any lateral tilt, rotation, or height discrepancies.

5. Knees:

- Adjust knee alignment for valgus (knees inward) or varus (knees outward):

- Use a yoga ball between knees for valgus.

- Use a resistance band to open knees outward for varus.

6. Feet:

- Squeeze a yoga block between the feet to maintain proper alignment.

Step 2: Establish Intra-Abdominal Pressure

1. Inhalation:

- Direct the breath into the space between the ribs and groin, allowing for backward and lateral expansion in the side waist.

2. Exhalation:

- Focus on lowering the ribs while keeping the lower abdomen engaged and taut.

Step 3: Correction Movements

1. Inhalation:

- Focus on directing the breath into the concave areas of the body.

- Gently lift and stimulate the concave regions to promote expansion.

2. Exhalation Adjustments:

- Counter-rotate any rib protrusions (rib humps).

- Adjust pelvic lateral tilt or height asymmetry.

- Exhale diagonally toward the rib hump, creating self-guided counter-rotation.

This step-by-step approach encourages spinal alignment, improved breathing mechanics, and muscular balance, especially targeting areas with asymmetry.

6. Standing Hip Push on a Stall Bar

Basic Standing Position Adjustment:

1. Setup:

- Position the stall bar on the opposite side of the pelvic shift.

2. Arm Placement:

- The hand on the concave side of the thoracic spine grips the bar diagonally.

- The hand on the convex side of the thoracic spine pulls in the opposite direction for traction.

Breathing Instructions:

1. Inhalation:

- Fully extend your body as you inhale.

- Direct the breath into the concave area of the thoracic spine.

2. Exhalation:

- Quickly exhale while focusing on breathing diagonally toward the rib protrusion side to encourage counter-rotation.

Pelvic Movement:

- Actively shift the pelvis toward the stall bar by pushing the hip against it.

This exercise aims to enhance spinal alignment, improve pelvic mobility, and promote targeted muscle activation on the concave and convex sides.
