# Supplementary material for: Effects of different physical therapy training protocols on patients with idiopathic scoliosis: Short-term results
Source: PLoS One. 2025 Oct 17;20(10):e0334713. doi: 10.1371/journal.pone.0334713 (PMC12533888; doi:10.1371/journal.pone.0334713)
Supplement: S2 File — (DOCX) [file pone.0334713.s002.docx]

**SPS group programs**

Based on the principles of Spiral Stabilization (SPS), a personalized training plan should be developed according to the patient's specific condition, with a total training duration of 30 minutes per session to ensure optimal rehabilitation outcomes. Below are the training principles and proposed training plan.

Spiral Stabilization Muscle Band Treatment for Scoliosis

-Adjustment of Peripheral Areas:

1. Correcting Peripheral Muscle Imbalances:

Training focuses on improving the balance and coordination of muscles in the shoulder girdle, pelvic girdle, and trunk to restore symmetry.

2. Establishing Muscle Bands:

Specific spiral stabilization exercises are used to activate spiral muscle chains, enhancing spinal support.

3. Gait and Walking Training:

Gait training is utilized to improve coordination and stability, ensuring that the spine maintains proper biomechanical alignment during movement.

4. Correcting Spinal Alignment:

Muscle bands are used in the central plane to straighten the spine and restore its natural physiological curves, such as cervical, thoracic, and lumbar curvature

-Adjustment of Central Areas:

1. Core Stabilization and Movement Patterns:

Correct movement patterns are established through core stabilization training to enhance strength and maintain proper spinal alignment.

2. Muscle Coordination and Balance:

Targeted training of the shoulder girdle, pelvic girdle, and trunk ensures balanced strength and flexibility.

3. Increasing Range of Motion:

Special attention is given to enhancing the backward movement range of the shoulder girdle, pelvic girdle, and trunk to optimize overall body mobility.

-Principles of Correct Movement:

1. Training in the Vertical Axis:

All exercises are conducted along the vertical axis of the torso to ensure spinal stability and symmetry.

2. Muscle Balance:

Balancing the muscles in the shoulder girdle, pelvic girdle, and trunk promotes overall body coordination.

3. Gait Optimization:

Repeated gait coordination training helps establish a stable and functional walking pattern.
