# Supplementary material for: Effects of different physical therapy training protocols on patients with idiopathic scoliosis: Short-term results
Source: PLoS One. 2025 Oct 17;20(10):e0334713. doi: 10.1371/journal.pone.0334713 (PMC12533888; doi:10.1371/journal.pone.0334713)
Supplement: S3 File — (DOCX) [file pone.0334713.s003.docx]

**Core stabilization group programs**

1. All Fours Exercise

Start on your hands and knees, with your shoulders stacked over your hands and your hips directly above your knees. This is called the four-point position. Draw in your tummy and ensure your spine is in a neutral position. From here exhale, activate your glutes and slowly extend your opposite arm and leg in a controlled manner. Slowly lower on the inhale to the starting position and then repeat on the other side.

30 seconds each time, switch sides, 5 times on each side, a total of 5 minutes.

2.Cat-Camel Exercise

On hands and knees, maintain tight abdominals with head straight (Photo 1). Take a deep breath in and lift your lower rib cage, round your back and relax your neck (Photo 2). As you breathe out, lower your chest towards the floor, looking slightly upward. Return to beginning position with tight abdominals.

Complete one movement in 30 seconds, do 10 times, a total of 5 minutes.

3. Single Leg Balance

With your eyes open, bend one knee up and balance on one foot. At first you may use your hands, like holding the back of a chair, table, or the wall. As balancing gets easier, take your hand(s) away and place them out to the side. Challenge yourself by bringing your arms across your chest.

Hold for 30 seconds Repeat on both legs, a total of 5 minutes.

4. Pelvic Tilts

Lying on back with knees bent and feet flat on the floor. Flatten back by tightening stomach muscles and buttocks. Hold for 10 seconds, breathing normally. a total of 5 minutes.

5. Double-Leg Abdominal Press

Lying on back with knees bent and feet flat on the floor, keeping your back in a neutral position. Raise your legs off the floor one at a time so that your knees and hips are bent at 90° angles.

Push your hands against your knees while pulling your knees toward your hands, which will engage your abdominal muscles. Keep your arms straight! Hold for three deep breaths.

Repeat __10__ times per set. Do __2__ sets per session. a total of 5 minutes.
